# Additive opportunistic capture explains group hunting benefits in African wild dogs

**DOI:** 10.1038/ncomms11033

**Published:** 2016-03-29

**Authors:** Tatjana Y. Hubel, Julia P. Myatt, Neil R. Jordan, Oliver P. Dewhirst, J. Weldon McNutt, Alan M. Wilson

**Affiliations:** 1 Structure and Motion Laboratory, Royal Veterinary College, University of London, Hatfield AL97TA, UK; 2 School of Biosciences, University of Birmingham, Edgbaston, Birmingham B15 2TT, UK; 3 Botswana Predator Conservation Trust, Private Bag 13, Maun, Botswana; 4 Centre for Ecosystem Science, School of Biological, Earth and Environmental Sciences, University of New South Wales, Sydney, New South Wales 2015 Australia; 5 Taronga Conservation Society Australia, Applied Eco-Logic Group, Taronga Western Plains Zoo, Dubbo, New South Wales 2830 Australia

## Abstract

African wild dogs (*Lycaon pictus*) are described as highly collaborative endurance pursuit hunters based on observations derived primarily from the grass plains of East Africa. However, the remaining population of this endangered species mainly occupies mixed woodland savannah where hunting strategies appear to differ from those previously described. We used high-resolution GPS and inertial technology to record fine-scale movement of all members of a single pack of six adult African wild dogs in northern Botswana. The dogs used multiple short-distance hunting attempts with a low individual kill rate (15.5%), but high group feeding rate due to the sharing of prey. Use of high-level cooperative chase strategies (coordination and collaboration) was not recorded. In the mixed woodland habitats typical of their current range, simultaneous, opportunistic, short-distance chasing by dogs pursuing multiple prey (rather than long collaborative pursuits of single prey by multiple individuals) could be the key to their relative success in these habitats.

## Abstract (web summary)

African wild dogs hunt collaboratively over long distances in grassland plains. Hubel *et al*. use high-resolution GPS to show that in their remaining habitat of mixed woodland savannah, African wild dogs use multiple opportunistic short-distance hunts, with no evidence of cooperative chasing.

## Graphical abstract

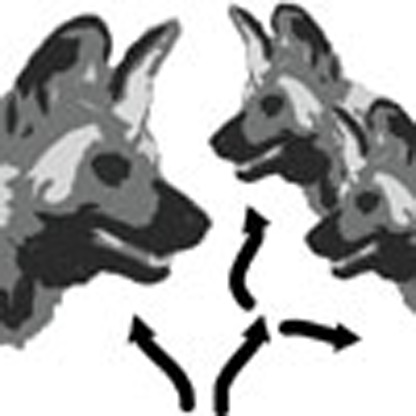

African wild dogs (*Lycaon pictus*) have been described as the ultimate cooperative persistence predator^1^,^2^,^3^ with packs reported to pursue prey over many kilometres^1^,^4^,^5^. This description is based on early observations of hunting in open, grass plains habitats such as those found in parts of East Africa. More recent and detailed knowledge of their range and distribution, including those in East Africa, shows remaining populations of African wild dogs are found primarily in woodland and woodland savannah habitats^6^,^7^,^8^, raising questions about the factors that facilitate their survival in these habitat and whether their hunting strategies differ significantly from earlier descriptions of hunting in open plains^9^,^10^,^11^.

Cursorial (running) hunting, exemplified by the highly athletic cheetah (*Acinonyx jubatus*)^12^, requires pursuit and out-manoeuvring of a prey animal to enable capture, and is energetically costly compared with ambush predation. Canids and lions (*Panthera leo*) are characteristically slower and less manoeuvrable than the cheetah, but are thought to maintain high kill rates by hunting in a group. The benefits of group hunting may include reduction in hunting distance, a higher kill rate and capture of larger prey^1^,^2^,^8^,^12^,^13^. Larger prey can provide greater returns per individual than smaller prey or feed more individuals, but their capture may require long, energetically costly runs and/or cooperation of multiple individuals to subdue them^3^,^6^.

In the African wild dog, group hunting has been reported to include higher-level cooperative strategies such as coordination (multiple individuals focus their attention on a single prey and relate to one another in space and time) and collaboration (multiple individuals chase a single prey, taking on different roles) as defined by ref. 14, modified by ref. 15 and reported in lions^16^.

We hypothesized that African wild dogs in mixed woodland savannah with comparatively low visibility, dense vegetation and ground cover would use hunting techniques that differ from the long-distance endurance pursuit of individual prey by multiple African wild dogs previously described for open grassland habitats.

We selected a focal study pack of free-ranging African wild dogs (hereafter referred to as ‘dogs') in northern Botswana consisting of six adults (Supplementary Table 1): a dominant male, an unrelated dominant breeding female and their two male and two female siblings. A litter of dependent pups was also present during part of the study period. This pack was selected from the nine extensively studied packs inhabiting the study area in contiguous and similar habitat as it was typical in terms of size and composition. Whilst only in our focal pack were all individuals collared, comparable locomotor data were obtained from an additional 18 collared individuals from a total of 13 other packs to obtain comparative data sets for a range of parameters indicative of hunting behaviour, for example, distance travelled per day, speed, acceleration and run distances. This was to ensure that results from this unique study were generally representative of the study population as a whole.

To investigate the assertions of high-level cooperative hunting based on extreme persistence, we deployed GPS–IMU (inertial measurement unit) collars on all adult members of our focal pack to record the fine-scale movements of all pack members simultaneously during hunting (Fig. 1). These high-resolution data loggers dynamically switch between different recording modes to acquire highly detailed data in response to acceleration. This enabled us to analyse hunting behaviour at the individual and group level. Prey species and behaviour were not recorded, but kill was inferred by feeding behaviour, and >80% of prey taken in the area are impala^9^,^17^. As the vocabulary relating to hunting has not previously been applied consistently, terms used in this study are defined in ‘Methods'. Here we define all locomotion in pursuit of food as hunting, with speeds above 3 ms^−1^ being described as ‘running', and as ‘chasing' when speed exceeds 6 ms^−1^, indicating a gallop gait. Since a key purpose of the study was to investigate whether our pack of dogs hunted collaboratively, we also examined whether a single dog was chasing (single-dog chase, SDC) while the remainder of the pack was not running or chasing, or multiple dogs were running simultaneously with at least one chasing (multiple-dog chase, MDC), timed from the start of the first dog reaching chase speed until cessation of running by all dogs in the group. Where an SDC or MDC was followed by an episode of feeding (determined from GPS data indicating the dogs remained in the same location for at least 5 min following a chase and other pack members converged to that location), the hunting event was classified as resulting in a kill.

The analysis here shows that members of the pack used short, opportunistic high-speed chases during hunting. We found no evidence of high-level cooperative chase strategies (coordination and collaboration) or of sustained pursuit of prey either by individuals or the pack. This result contrasts with descriptions of highly cooperative, long-distance endurance pursuits from the short grass plains of East Africa and is likely to be more representative of African wild dog hunting behaviour in the woodland habitats typical of their current range and may account for their continued survival in these habitats.

## Results

### Hunt description

A cumulative total of 1,551 runs (maximum stride speed >3 ms^−1^) was recorded from the six adult pack members. If a run contained a stride when speed exceeded 6 ms^−1^, the run was further classified as a chase (*n*=1,119). A hunt was defined as encompassing the time and distance covered by an individual in search of prey and ending in a high-speed chase, with a mean distance per chase (+s.d.) of 445.5±30.1 m (median: 316.6 m (Q1=311.3 and Q3= 337.1)). Direct observations of the pack were made to validate movements recorded by the collars, including the typical pattern of travelling together (low spatial dispersion) at comparatively low speeds (walking and trotting) presumably until prey were flushed, followed by one (SDC) or more dogs (MDC) accelerating to higher speeds. When analysing certain aspects of group hunting behaviour, single- and multi-dog chases were combined as dog chases (DC=total of MDC and SDC).

Each individual chase was initially analysed in isolation to determine chase distance, duration, maximum stride speed, maximum tangential (fore-aft) acceleration and deceleration, maximum centripetal acceleration (turning, right and left), mean absolute heading rate and tortuosity. Chases were subsequently categorized based on the number of runs occurring simultaneously. The chases were then re-analysed in the context of group dynamics by analysing each MDC incorporating all simultaneous runs (from the time the first dog started running to when the last dog stopped running).

Excluding chases near the den site, movement data yielded (mean+s.d.) 2.43±0.88 chases per individual per day and 5.5 MDCs per pack per day, a value similar to published data^6^ (4.2 chases per pack per day).

### Comparison to other packs

The range and daily distance travelled by the focal pack was comparable to the mean of the 13 other packs in our study. We assumed that daily distance travelled by each dog was representative for their pack. The mean daily distance travelled by the dogs of the focal pack was 13.2 km and by the dogs of all other packs was 13.8 km. Run data for four individuals from three different packs were analysed and grouped together. Both groups (focal pack, individuals from other packs as other group) had maximum stride speeds of 19 ms^−1^, and reached tangential and centripetal accelerations of at least±8 ms^−2^. Chase distances for the other dogs were slightly lower, most likely due to the different collar-triggering method (Supplementary Fig. 1).

### Hunting strategy and kill rate

Data were parsed into run sequences defined by a series of GPS locations and animated (examples in Supplementary Movies 1 and 2), allowing a detailed visual analysis of each dog's position in relation to the other pack members. Higher levels of cooperation require individuals to target the same individual prey, and to relate to one another in space and time^14^,^15^, either by chasing together (coordination) or assuming different but complimentary roles (collaboration^15^). In this pack we found no evidence for cooperation beyond travelling together and sharing prey. Forty per cent of MDCs involving three or more individuals show a dispersion of most or all animals in multiple directions with no spatial relationship, and while the remaining 60% show multiple dogs running in the same general direction (Supplementary Movies 1 and 2), there was no clear evidence of specific roles (for example, dogs blocking or running while converging from different directions). Prey information is unavailable but the vast majority of runs did not end with all dogs at the same location, as would be expected when attacking the same prey.

Hunting outcome was analysed based on (1) individual kill rate (kill to chase ratio) and (2) on a group basis when one or multiple dogs ran simultaneously (DC) and at least one kill occurred. Because direct observations of kills were infrequent, kills were inferred from analysis of animations by the pattern of post-capture movement and subsequent convergence by the remaining pack members: a chase (independent of number of dogs) that ended at a (non-den) point, where the remainder of the pack regrouped (within 50 m of the end of one of the chases) for at least 5 min (approximate minimum time to consume a small antelope based on field observations; very small prey may not be detected). On the basis of these criteria, 116 DC were identified as kills (at least one kill among all dogs running) over 104 days resulting in an average of 1.16 kills per day. An individual kill rate of 0.155 was calculated based on number of hunts and kills conducted by each individual.

**Figure 2:**
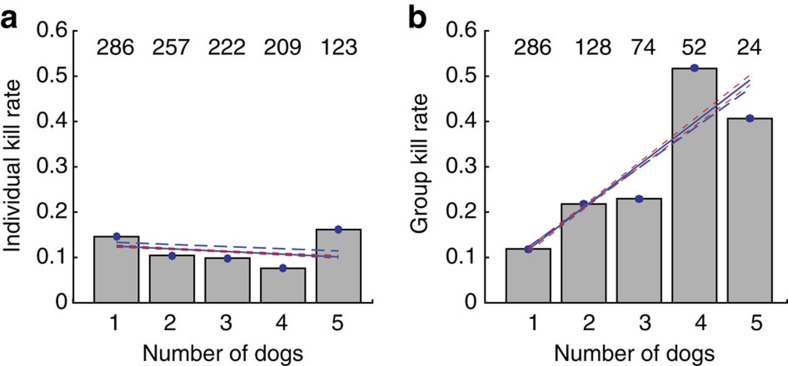
Relationship between kill rate and number of dogs running simultaneously. (**a**) Kill rate for individual chases and (**b**) group kill rate (DCs). (**a**) Number of chases identified as ending in kill divided by total number of chases within each group size (chases evaluated automatically as independent event, kill assumed if displacement of the dog conducting the chase is <50 m 5 min after the end of the run; total number of chases analysed, *n*=1,097). (**b**) Number of kills in DC (could be more than one in a DC) divided by total number of DCs within each group size (kills identified manually by four reviewers (at least three had to agree); total SDCs, *n*=286; total MDCs, *n*=278). Regression line (dashed blue line), weighted regression line based on number of observations in category (solid blue line) and curve fitting confidence interval to weighted regression line (dashed red line). Number of chases analysed in each group size displayed above histogram. Note: results in group size of 1 in **a** and **b** are theoretically identical (difference due to manual and automatic classification), and there were no successful chases for a group size of 6.

There was no evidence that individual kill rates increased in MDC settings (Fig. 2a, analysis of variance, *n*=5: *p*=0.191). Group kill rate increased significantly with group size (Fig. 2b, analysis of variance, *n*=5: *p*=0.029), but not beyond the level expected when multiplying the individual kill rate by the number of dogs running simultaneously.

### Run parameter and group size

**Figure 3:**
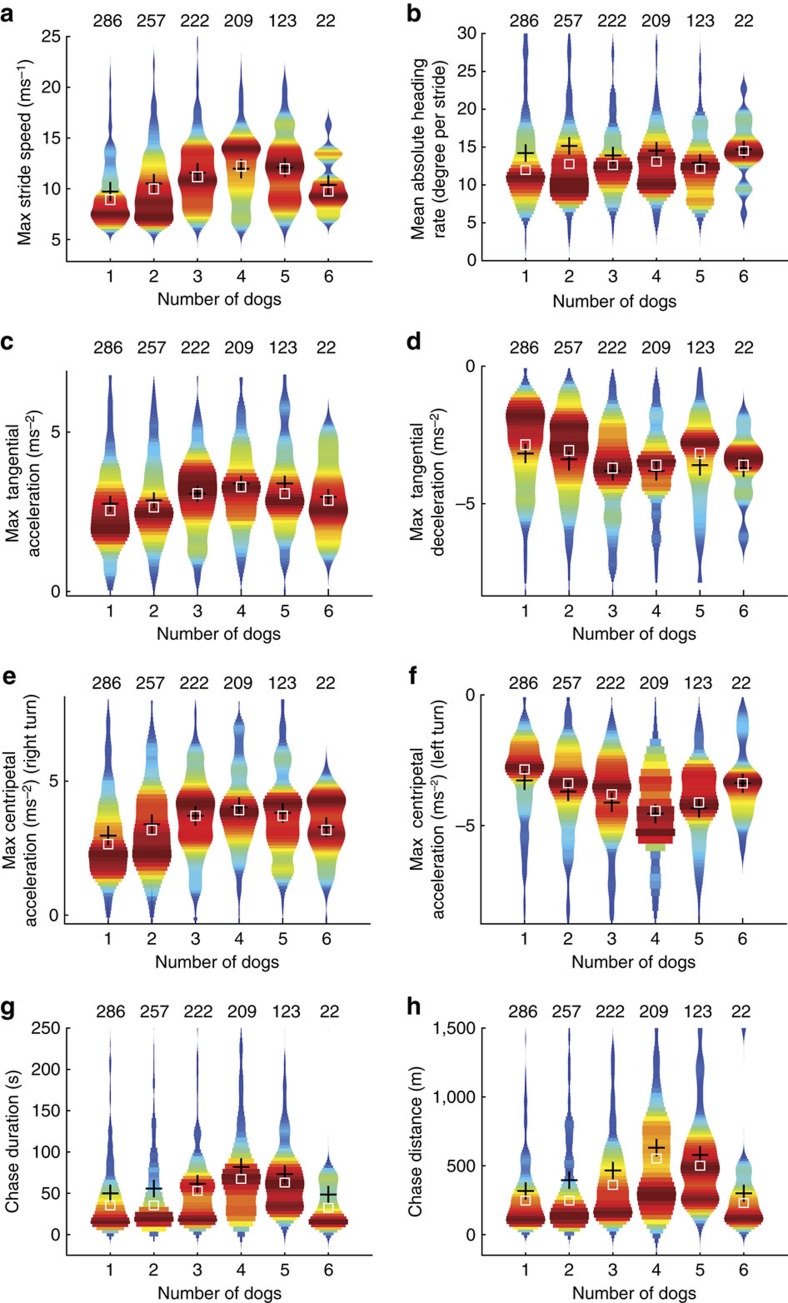
Chase parameters versus the number of dogs running simultaneously (group size) displayed as violin plots (combining box plot and kernel density plot). Number of chases analysed, *n*=1,119. Violin plots show the density distribution of the values, with each histogram normalized to the same maximum bin width compared with the distribution shape. The total number of values contributing to each histogram is given above each plot, mean (black cross); median (white box). (**a**) Maximum (Max) stride speed, (**b**) mean absolute heading rate (degree per stride), (**c**) maximum tangential (fore-aft) acceleration and (**d**) deceleration, maximum centripetal (turning) acceleration (**e**) turning right and (**f**) turning left, (**g**) chase duration and (**h**) chase distance.

**Figure 4:**
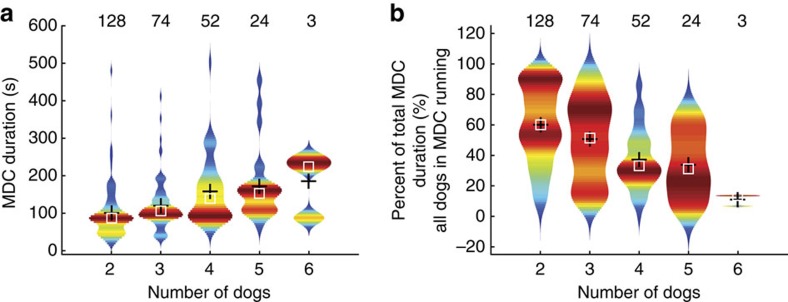
Total duration and simultaneous run duration of MDCs. (**a**) Total MDC duration versus number of dogs involved (time from when first dog starts running until last dog stops running). (**b**) Percentage of time during which all dogs participating in MDCs are running simultaneously. Number of runs in each group size displayed above each kernel histogram: total number of MDCs, *n*=278; total number of runs in all MDCs, *n*=382.

The relationship between run parameters (maximum stride speed, tangential acceleration/deceleration, centripetal acceleration (right and left), heading rate, run duration and run distance) and group size (number of dogs running simultaneously) was analysed for three sub-data sets to test for robustness: all runs (that is, >3 ms^−1^), chases >6 ms^−1^ (that is, only chases/hunts) and successful chases only (Fig. 3 and Supplementary Fig. 5). Violin plots of maximum stride speed, maximum tangential (fore-aft) and centripetal acceleration (right turn), distance and duration of runs all increased with number of dogs participating up to four dogs (Fig. 3a,c,e,g,h), while maximum tangential deceleration and centripetal acceleration (left turn) decreased (Fig. 3d,f). No clear trend was visible in tortuosity (total distance travelled divided by straight-line distance from beginning to end of a run) or the average change in heading per stride over the run (mean absolute heading rate, alternative metric to centripetal acceleration for manoeuvring; Fig. 3b). Duration of individual chases (either SDC or as part of MDC) increased with number of dogs involved up to four (Fig. 3g) with an average duration of 60.8±5.1 s (mean±s.d.). The duration of MDCs also increased with group size (Fig. 4a). However, the period when all dogs were running was typically brief, contrary to what would be expected with coordinated group hunting, for five dogs it averaged 34.0% of the total MDC time (Fig. 4b and Supplementary Movie 1).

The relationship between group size and the dependent parameters was evaluated using a multivariate general linear model (GLM). Although statistical results supported the relationships described above, there was considerable co-variation between the parameters, limiting the weight that should be attributed to this analysis. (Supplementary Table 2).

### Run participation and initiation by individuals

**Figure 5:**
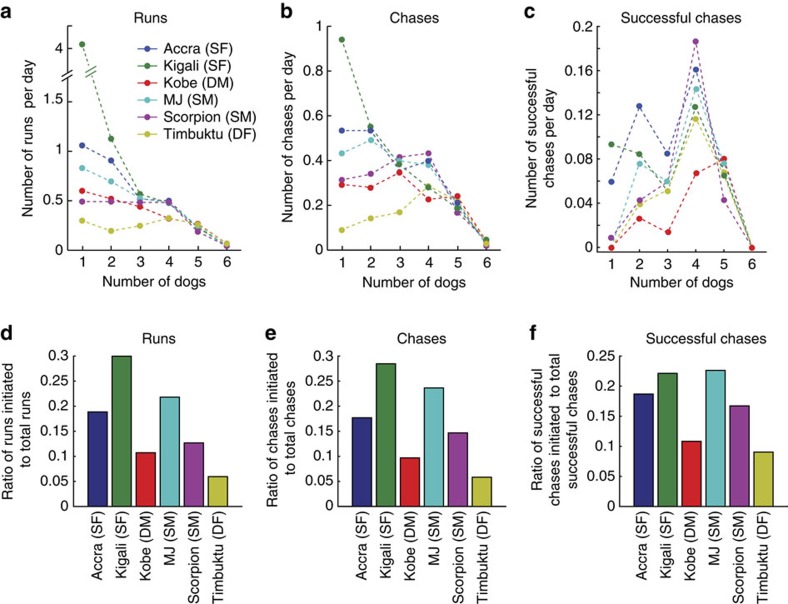
Run participation and initiation. (**a**–**c**) Number of runs/chases each dog (colour key in **a**) executed in each group size (**a**) for runs (>3 ms^−1^, *n*=1,551), (**b**) chases (>6 ms^−1^, *n*=1,119) and (**c**) successful chases (resulting in kill, *n*=127). (**d**–**f**) Fraction of time individual dogs started to run first in MDC for (**d**) runs, (**e**) chases and (**f**) successful chases. All data (**a**–**f**) normalized by number of days the individual dogs were available for hunting. DF, dominant female; DM, dominant male; SF, subdominant female; SM, subdominant male.

The number of runs and chases each individual participated in (Fig. 5a–c and Supplementary Note 2), as well as the number they initiated (Fig. 5d–f), was adjusted to account for the number of days an individual was available to hunt—inter-individual variance due to, for example, mortality (Kobe), and confinement at a den (Timbuktu). The dominant individuals were less likely than the subdominant individuals in the pack to initiate (binomial test of proportions, *n*=1,119: *χ*^2^(1)=8.7922, *P*=0.003) and participate in MDCs (*χ*^2^(1)=40.7126, *P*<0.001).

### Spatial relationship between pack members

**Figure 6:**
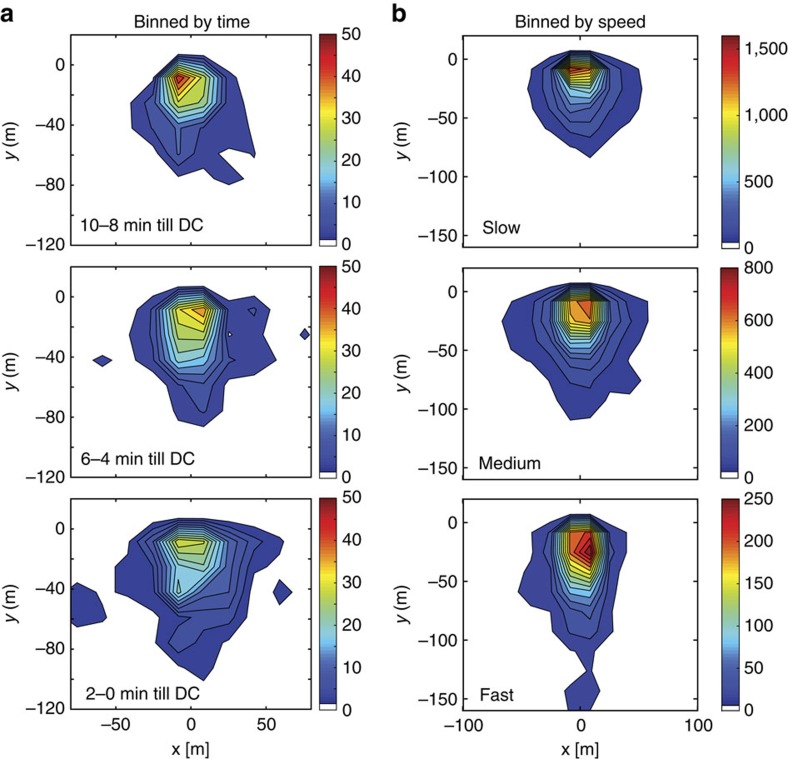
Group formation pattern before onset of chase. Spatial relationship between individual dogs in the 10-min leading up to the beginning of a DC (SDC or MDC) as a function of (**a**) time and (**b**) speed. The three-dimensional location histograms show the position of the other group members, with respect to the individual leading the pack. The colour scale is the count of how often any given dog was present at a certain location. Analysis based on DCs occurring during the 2 h of 10-s GPS data sample rate during the daily main hunting period (DCs; *n*=100). In **a** position of the individuals was averaged over 2-min periods for the 10-min leading up to the DC and displayed for three out of five instances. In **b** 10-min data were binned by speed: slow speed, 0–1.5 ms^−1^; medium speed, 1.5–3 ms^−1^; and fast speed, >3 ms^−1^.

Our data show that MDCs have no discernable pattern or organization. The location and movements of individual dogs are likely to be determined by the location, speed and heading of prey encountered. To determine whether more coordinated movement occurred when the pack travelled at lower speeds, we analysed the spatial relationship between pack members over the 10 min before the start of all DCs. Moving in a specific characteristic formation could aid prey detection (spear-shaped or parallel formation), minimize visual exposure (travelling in line) or assist in surrounding prey (assume a U shape). A rotation matrix was applied to each point in time to reorient individuals in the centroid heading direction; the lead animal was identified as the one furthest ahead in centroid heading direction and used as the point of origin. We investigated movement patterns according to their occurrence in the timeline heading up to the run, as well as based on speed classes. Figure 6a shows heat maps at time points before a DC has been initiated, illustrating that dogs travel in no particular formation but at a slightly wider distribution (more spread out) shortly before starting a DC. When averaged within speed bins (Fig. 6b) the heat maps show a narrower cluster for slow speeds. Individuals travel at a mean (±s.d.) distance of 42.8±42.7 m (median: 26.6 m (Q1=14.20 and Q3=54.9)) from the centroid.

**Figure 7:**
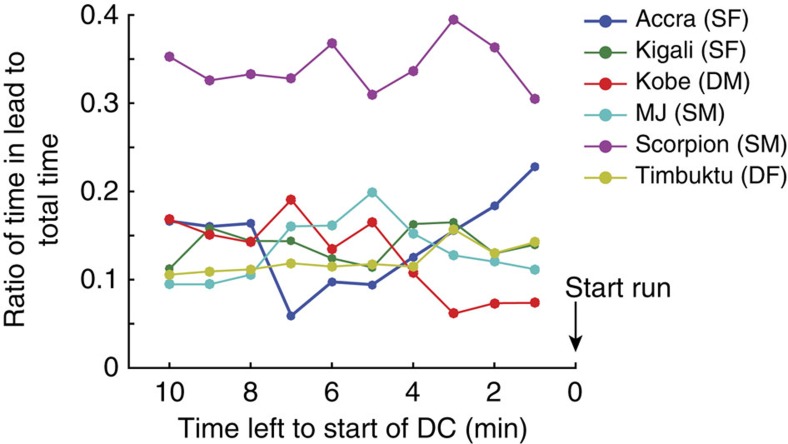
Leadership based on spatial position between chases. Ratio of time individual dogs are in the lead to total time leading up to DC, normalized by number of days each individual was available for hunting. Lead defined based on pack centroid position and heading. Abscissa shows time left leading up to the beginning of the DC. Analysis based on DCs occurring during the 2 h of 10-s GPS data sample rate during the daily main hunting period (DCs; *n*=100). DF, dominant female; DM, dominant male; SF, subdominant female; SM, subdominant male.

Using the Hodges–Ajne test for directional data analysis we determined a preferred position in fore-aft direction for three individuals (Methods and Supplementary Fig. 7). One individual (Scorpion, a subdominant male) led more often than others (Fig. 7).

### Influence of vegetation on chase outcome

We tested whether vegetation influenced chase outcome^13^ using aerial photography images (source Google Earth) and classifying the percentage cover of four vegetation/habitat types (modified from ref. 18) at the beginning and end of each chase (Methods). Whilst there was significantly more scrub vegetation at the end of chases compared with the beginning (*P*<0.034; Supplementary Table 3a), the difference was slight (1%) and was considered unlikely to have an influence on hunt outcome. There was no significant correlation between vegetation cover and kill rate (Supplementary Table 3b).

## Discussion

We present unique data on the fine-scale relative position, speed and activity of all individuals in a pack of African wild dogs during hunting. These data allow, for the first time, insights into group hunting behaviour not only in unprecedented detail but also in an area of dense vegetation, which rarely permits direct observations of hunting: in 3 months of direct follows in the field, only a single kill was directly observed to completion (see Supplementary Note 1 for comparison of observation and GPS recording).

African wild dogs have been described as coursing predators, chasing their prey over long distances and exhibiting elaborate collaborative strategies^6^,^10^, including relay running^4^,^5^ or spatial distributions that reflect higher-level cooperation (that is, coordination and collaboration)^1^,^3^.

We found no evidence of cooperative hunting in our pack beyond the greeting and pre-movement ‘rally' behaviour suggested to promote pack cohesion before hunting^17^, the travelling of the pack together while hunting (during which all individuals partake in chases at some stage) and the active sharing of kills.

We found no evidence of clear spatial patterns when dogs were moving between chases, such as a line, U or spear formation, that could aid in the detection or capture of prey. The spacing we found (median distance from centroid±26 m) should contribute to increasing encounter rate of prey flushed due to greater area coverage by spatially dispersed dogs than if moving as a tight cluster or single file (Fig. 6).

Whilst individuals did not take on distinct roles during the chases, individual roles while hunting did vary. There were individual preferences for position along the fore-aft direction of travel. ‘Scorpion' (subdominant male) led the pack significantly more often than any other individual, while ‘Kigali' (subdominant female, with a healed fore-leg injury that caused her to limp), spent more time at the rear of the pack. Dogs also differed in their MDC initiation rate. Although Scorpion lead the pack more than twice as often as would be expected by chance (Fig. 7), he initiated fewer MDCs than some others (Fig. 5d–f). There were also differences in how often each individual participated in MDCs. The dominants (Kobe and Timbuktu) initiated and participated least in MDCs. This might be expected for the dominant female based on her pregnancy and unique role in reproduction; it has previously been noted that dominants do not always lead hunts^17^,^19^. Dominants typically retain priority access to feed at kills^20^, while the oldest subdominants feed last. Consequently, subdominants may be more likely to be motivated to hunt and to arrive early at a kill to feed before dominants and/or pups arrive (for example, ref. 20).

Cooperative hunting and increasing group size have been reported to increase rate of kills, reduce costs per individual and/or enable capture of larger prey^6^. Prey size and group kill rate were found to correlate positively with pack size (4–20 adults) and negatively with chase distance of large prey (up to 200 kg)^6^. In contrast, our results show speed, tangential and centripetal acceleration, and chase distances all increased with pack size up to four adults, above which these parameters remained constant or declined slightly. Values of these parameters declined in this study for group sizes greater than four, but this could be explained by reduced performance of two individuals: two females, ‘Kigali' and ‘Timbuktu', were constrained by injury and heavy pregnancy, respectively, and in this pack of six adults, one or both were included in all MDCs with more than four participants, while MDCs with fewer participants could exclude both of them, diminishing their influence. This interpretation is supported by the fact that forced removal of individuals in the analyses diminishes the effect (Supplementary Fig. 6). An increased number of dogs running could reflect higher motivation among those individuals, or a perceived better opportunity to capture prey, or simply a tendency to run and chase when other dogs are doing so.

In our pack, group kill rate correlated positively with group size, but not beyond what would be expected when multiplying a constant individual kill rate by the number of individuals involved in the MDC (Fig. 2).

Identification or size of captured prey species could not be inferred from logged movement data of dogs in this study. However, in this population^21^, as well as the two other most-studied African wild dog populations, more than 80% of prey are the most abundant medium-sized antelope species^9^,^17^. Since impala are the predominant prey in our study area and considering the time of the study (April–October), this would give a possible variation in impala size between 20 and 60 kg at the beginning of the study (juvenile–large adult male) and subadult to adult by the end. Prey of this size can feed multiple dogs up to a certain pack size with little or no diminishing returns to any individual^21^. Therefore, in our pack, the benefits derived from group hunting described by Creel and Creel^6^ and Fanshawe and Fitzgibbon^10^ can be explained simply by additive opportunistic capture frequency, as expected when more dogs chase multiple individual prey, and feed cooperatively.

In our focal pack of six dogs, chase parameters including chase distance increased with number of dogs hunting in MDCs (Fig. 3). Previous studies report reduced hunt distance with larger packs, which may reflect those pack sizes or prey choice^6^.

Creel and Creel reported 75% of chases that end in denser vegetation resulted in kills, versus 37% for chases in more open or more uniform vegetation. We found vegetation density made no significant difference to kill rate, nor evidence that the dogs drove or directed prey towards more dense vegetation.

Our study was conducted in an extensive wildlife area characterized by a mosaic of habitat types but dominated by mixed woodland. This is consistent with the habitats of most of the remaining populations of this endangered species. The focal pack was comparable to mean adult pack sizes (4.8–8.9 adults) described in five different ecosystems^6^ and home range, and distance travelled per day was similar to other packs in the study area. Our pack demonstrated that opportunistic hunting with no collaboration is a successful strategy in mixed woodland savannah with an abundance of medium-sized prey. While no other pack was collared in entirety, data from additional similarly collared individuals in other packs in the same study area showed the same patterns of movement and hunting with short, fast runs interspersed by distances travelled at lower speeds, indicating this to be a typical hunting strategy.

This pack of six adult African wild dogs captured prey by performing multiple short, high-speed chases interspersed with travelling through their range at walk and trot. Hunting was characterized by multiple, short-distance chases, with increased group kill rate proportional to the number of dogs running simultaneously, and through sharing of prey. Moving as a moderately dispersed group might aid prey detection, flushing and capture, but frequent use of higher-level cooperative chase strategies (coordination and collaboration) was not recorded. The endurance/persistence and cooperative hunting behaviour of African wild dogs has been a recurrent theme in literature since the nineteenth century^22^,^23^,^24^. Detailed descriptions of such from the short grass plains of East Africa in the 1970s are in stark contrast with results from this study in the mixed woodland and woodland savannah habitats that form the majority of their extant range. The opportunistic hunting strategy consisting of multiple short, high-speed chases of multiple medium-sized prey rather than long-distance, high-investment pursuit of larger prey might contribute to their relative success in these habitats.

## Methods

### Animals

The packs in this study were located in the Okavango Delta region of Northern Botswana and are part of an ongoing study by Botswana Predator Conservation Trust (http://www.bpctrust.org). Every member of a pack of six adult dogs (focal pack) was collared. The pack consisted of a dominant male (Kobe) and a dominant female (Timbuktu), two subdominant males (MJ and Scorpion) and two subdominant females (Accra and Kigali). Data collection on all pack members started on 13 April 2012 and continued over the following 5–7 months, with one collar failing on 27 May. The collar was replaced, but the failure resulted in a lack of data for one dog (Accra) over a period of 22 days. This time period was removed from our analysis. Collar removal started at the end of August 2012. One dog (Kobe), the dominant male, died on 27 June. The data from the dominant female (Timbuktu) show a period of low activity when she remained at the den with pups. Distance travelled per day was calculated excluding the denning female Timbuktu.

The dogs were immobilized by free darting from a vehicle using xylazine (55 mg), ketamine (50 mg) and atropine (1.1–1.2 mg), and reversed after 45–60 min, with yohimbine (4 mg) or atipamezole (5.5 mg). While sedated, anatomic measurements including limb lengths, limb and body girths and body mass were recorded (Supplementary Table 1). Collar data were retrieved via radio link to a ground vehicle every few weeks.

*Comparison to other packs*. To demonstrate that our focal pack is representative of all the packs in the area we used high-resolution GPS collar data from 18 subdominant individuals from 13 different packs in the area. The collars worn by African wild dogs outside the focal pack were either the same as or an earlier version of the collars used on the focal pack. Outside the focal pack data were recorded at 1-h intervals when dogs were resting, and at 5- or 10-min intervals when they were moving. Only four collars were allowed to go into ‘run state' for a limited trial period not exceeding a total of 2 months. Data were collected for time slots of different duration (21–409 days) between November 2011 and October 2014.

To compare the focal pack with the individuals from other packs we compared daily distance travelled and chase performance. For daily distance travelled we calculated the mean±s.d. of the individual medians in the focal pack and compared it with the group containing all individuals from other packs. Maximum speeds and accelerations are extracted from histograms of the respective stride parameter in both groups (Supplementary Fig. 1).

### Collar design and data recording

Power consumption poses a major challenge in the design of a wildlife tracking collar. To fulfil the demands of sufficient data rate during periods of high animal activity and average low energy consumption we used collars designed in-house and previously used successfully on cheetahs^12^. The collars use in-built solar cells on the top housing and careful management of the GPS sample rate for power conservation. The mass of the mark 2 collars was ∼340 g. Dropoff units (Sirtrack; 70 g) were used to release two collars at the end of the study. Other collars were removed following immobilization.

The collar was controlled by a low-power MSP430 16-bit microcontroller (Texas Instruments Inc., TX, USA), running custom software written in the ‘C' programming language. A 2-GB micro-SD flash memory card (Sandisk, CA, USA) was used for on-board data storage.

The collar provides GPS position and instantaneous velocity data, as well as three-axis specific force and rotation rate data. GPS position and velocity were obtained from an LEA-6T GPS module (u-Blox AG). An MMA7331 three-axis accelerometer module (Freescale Semiconductor) provided specific force with a ±12-*g* range. The roll and pitch rotation rate was measured by a dual-axis gyroscope (ST Microelectronics), and yaw rotation rate by a single-axis gyroscope (ST Microelectronics), both set to the 2000 degree per second range. Sensor outputs were filtered by simple single-pole analogue filters (100 Hz knee), and then sampled by the microcontroller at 300 (accelerometers) or 100 (gyroscopes) samples per second. Data download from the collar was via a 2.4-GHz chirp-spread-spectrum communication module (Nanotron Technologies Gmbh). Power was provided by two batteries. A 900-mAh lithium-polymer rechargeable battery (Active Robots), charged by a solar cell array consisting of 10 monocrystalline silicon solar cells (Ixys Koria), and a 13-Ah lithium thionyl chloride battery (Saft). The microcontroller measured both battery voltages and the charge current from the solar cell array and switched the collar electrical load between batteries depending on the battery state.

To manage power consumption effectively, the collar was programmed to switch dynamically between four different operating ‘states' (Supplementary Fig. 2). The state depended on the time of the day and the animal activity level (measured using the accelerometers). The different states enabled power rationing between average power consumption on the one hand, and quantity and resolution of data on the other. Multiple software updates were installed on the collars (remotely) during the research period to improve performance and capture as many hunts as possible. The default state (alert state) provided GPS positions every hour, and allowed the transition into ‘mooch state' with 5-min fixes when the animal was deemed active, based on periodic specific force measurements (measurement taken for 10 s at 30 Hz every minute). Initially, the collar was set to ‘ready state' when the animal was moving between local times of 18:00 and 20:00, since previous work suggested that most hunting occurs around dawn and dusk^25^. In ‘ready state' GPS positions and speeds were recorded every 5 s, if the animal was deemed to be active. A transition occurred from ‘ready' state to ‘run state' if fore-aft accelerometer data exceeded a threshold equivalent to galloping in three consecutive peaks, and the run was defined as valid and stored if five further peaks were detected. In ‘ready state' accelerometer data were recorded into a circular buffer at 100 Hz, the buffer storing the latest 3 s of data. This pre-buffering allowed open-loop inertial navigation back to the beginning of the run. However, it was later deemed that an extended time allowed for entering ‘run state' was more beneficial than the pre-buffering of data. Pre-buffering was abolished on 26 April 2012; this resulted in the loss of the first one or two strides at the beginning of the run. From then on the collar was allowed to enter ‘run state' directly from ‘mooch state' during pre-selected ‘times of interests' between 4:00 to 10:00 and 17:00 to 22:00 local time. During the ‘times of interest' GPS data were recorded every 5 min (the same as during normal ‘mooch state'), but sample rates were increased to every 10 s for a 2-h window within the ‘times of interest' to get a more accurate account of position during times when most hunts were expected to happen based on initial data observations. Initially, this time was chosen to be between 18:00 and 20:00 and later changed to between 06:00 and 08:00 local time.

### Signal processing

GPS data with horizontal position accuracy above 8 m were removed for all calculations.

In the ‘run state', the power management features used gave different sampling rates for accelerometer (300 Hz) and gyro (100 Hz). GPS position (5 Hz) and instantaneous velocity (5 Hz) were usually (but not always) available within 1 s after entering the ‘run state' but often not accurate until 4–6 s later (Supplementary Fig. 3c–f).

To reduce noise, improve precision and increase temporal resolution in the position and velocity data (Supplementary Fig. 3a,b), GPS and IMU measurements were fused as previously described^12^ using a 12-state extended Kalman filter^26^ followed by a Rauch–Tung–Striebel smoother^27^ written in MATLAB (The Mathworks Inc., MA, USA).

### Definition of locomotion

There is no global definition of the terms hunting, hunt or chases, and in the context of this study we define the terms as followed: hunting is all locomotion in the pursuit of food and encompasses multiple (mostly unsuccessful) hunts. A hunt is the locomotion in search (slow speed) and pursuit of a prey individual ending in a high-speed run (chase). We realized that some terms used might require a more extensive explanation due to the two-level analysis carried out to look at individual and pack performance. Terms such as ‘hunt', for example, can be applied to an individual or the pack. At the pack level, it is often defined as the time from the end of one group chase to the end of the next group chase (group hunt). Since not all individuals necessarily participate in a group chase, we defined hunt on an individual basis, encompassing the time and distance from the end of one chase to the end of the next chase by the same individual. A hunt encompasses a slow-speed (search) and a high-speed (chase) phase. Run were recorded at times when the collar went into high sample mode (based on an exceeded acceleration threshold) and maximum stride speeds exceeded 3 ms^−1^. Chase are all runs containing maximum stride speeds exceeding 6 ms-1 (galloping). We assume all chases to be in pursuit of prey. SDCs were instances when one dog was conducting a chase and no other dogs were running simultaneously. MDCs were instances when multiple animals run simultaneously, encompassing the time from the start of the first dog running till the last dog stopped. MDC included at least one chase (6 ms^−1^), but group size and duration is determined by number of animals running not necessarily chasing. This term accounts for pack activity during hunting. Individual kill rate was calculated by the number of chases ending in a kill vs the total number of chases by that individual, determined automatically from the number of times the dog stays at the end of chase position for five minutes or longer (indicating feeding). Group kill rate was determined manually from animations, the number of times any individual in the group made a kill vs number of MDCs for each group size. We define group hunt as a pack term, covering the period when one or more dogs are searching for prey at low speed with a subsequent MDC or SDC. It begins at the end of either an MDC or an SDC and ends at the end of the next MDC or SDC (Supplementary Fig. 4).

### Data analysis

The recording at high sample rate was triggered by the IMU and continued as long as the horizontal acceleration threshold was exceeded within a 5-s window. Overrun times between 5 and 20 s were implemented depending on the software update. Recordings at 5 Hz were restricted to 87 s, and runs exceeding this time while still showing speeds above 3 ms^−1^ were reconstructed based on 10-s data. We were unable to reconstruct the ending of 5.7% of the runs and assigned an ending randomly chosen out of the pool of reconstructed endings assuming the distribution is representative for all runs exceeding 87 s. Eighty per cent of the runs lasted <87 s and only a few (2.4%) lasted significantly longer. The difference in median distances covered per run between reconstructed and non-reconstructed data was 2.7%.

Recorded activity lasting <5 s and never exceeding 3 ms^−1^ (instantaneous GPS velocity) was excluded from the analysis leaving a total of 2,026 runs to be analysed; 69 runs failed to produce converged Kalman-filtered results (speed going towards infinity) and were removed. Sufficient strides (at least three per run) were successfully extracted from 1,641 runs. In 4% of the cases (65 runs) a second run was triggered within 30 s of the first ending and the two recordings were classified as a single run. The two runs were combined by linear interpolation of position and hence speed to fill the gap between them. In all, 1,551 runs (140,141 strides) contained at least one stride whose average speed exceeded 3 ms^−1^ (a speed determined to be slow canter). Runs exceeding a 6-ms^−1^ (galloping) stride speed threshold were classed as chases. We recorded 1,119 valid chases.

Chases were analysed with respect to their maximum stride speeds, maximum centripetal accelerations, maximum tangential accelerations/deceleration, heading rate, duration and distance covered. To present these results in the context of group hunting, run settings were categorized into SDC and MDC (two or more dogs running simultaneously; no spatial criteria). MDC include at least one dog chasing (>6 ms^−1^), but other dogs only needed to run (>3 ms^−1^) to contribute to group size. An MDC began when the first dog started to run and ended when the last dog participating in the MDC stopped (Supplementary Fig. 4).

### Kill rate and group behaviour

To visualize group behaviour (including those at lower speeds) we animated 791 instances of 1 dog running, 194 of 2 dogs, 93 of 3 dogs, 61 of 4 dogs, 24 of 5 dogs and 5 of 6 dogs running. These instances included 286 SDC and 278 MDCS.

Individual kill rate was assessed based on the number of kills versus the total number of chases by an individual dog. Group kill rate was based on number of kills observed at times when multiple dogs were running versus total number of instances with multiple dogs running. Both individual kill rate and group kill rate were calculated for different group sizes.

*Automatic identification of individual kills (individual kill rate)*. Individual chases were automatically classified as successful (ending in kill) if the dog remained for at least 5 min within a 50-m radius of the end of the chase. It was not possible to reliably classify feeding from labelled accelerometer data as previously done for cheetah^12^,^28^. Individual kill rates for chases within the context of their group setting were displayed in Fig. 2a. Individual success rate was calculated for each dog and averaged over all individuals, yielding a success rate of 0.155.

*Manual identifications of kills in group settings (group kill rate)*. Runs were displayed, once as a close up (Supplementary Movie 1) and once where each run sequence (of GPS locations) was extended to include 10 min before and after a run and the location of all dogs in the pack (regardless of whether or not they were also running; Supplementary Movie 2, examples). Four of the authors (T.Y.H., J.P.M., N.R.J. and J.W.M.) assessed each running event and scored it as successful or not (at least one of the dogs made a kill). These assessments were based on >25 years of collective field experience of African wild dogs in the study area. The criteria of a kill being made was identified as a run that ended at a point where other members of the pack subsequently regrouped (about ±50 m) and remained for at least 5 min following the end of the run. Events were classified as a kill if at least three of the assessors agreed. Group kill rates in relation to group size are displayed in Fig. 2b.

### Manual identification of other group behaviour

Further typical behaviour could be identified.

*Rally and greets*. The rally/greets could be identified by (1) the scale of the runs: within a small area, typically (perhaps at most) a 50 × 50-m area; (2) by numerous sharp turning movements around small areas within the total area; and (3) by other dogs joining and doing much the same general movements—remaining for 10 min in the small area where the rally/greet took place.

*Recruitment*. A recruitment, where dogs joined others at a site where a kill had already been made, is characterized by a near-direct run to a location where other dogs are already present (the site of a kill).

Examples of animation of unsuccessful runs, successful kills, recruitment and greetings can be found in Supplementary Movie 2 and of a detailed analysis of an observed kill and the corresponding animation in Supplementary Note 1.

### Daily distance travelled

Data collected under the different collar states were combined onto a single timeline to determine distance covered per day. Mean speed of each dog when moving slowly was taken as the straight-line distance/time between 5-min GPS fixes so is an underestimate if a tortuous route was followed.

### Calculation of speed and stride frequency

All data analysis was carried out using MATLAB. Fore-aft acceleration was used to determine stride peak times and stride frequency. A band pass Butterworth filter (fourth order) was applied with cutoff frequencies of 1 and 8 Hz, and assuming a maximum stride frequency of 3 Hz a peak detection function was used to detect peaks with a minimum duration of 0.33 s between peaks and a minimum peak height of 0.5 g. Maximum horizontal stride speed was derived from the Kalman-filtered and smoothed velocity averaged over strides.

### Calculating change of heading and tangential and centripetal acceleration

Mid-stride times were used to calculate tangential (fore-aft) acceleration, centripetal (turning) acceleration and change in heading between strides. The displacement vectors between consecutive strides were then calculated:


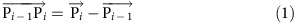


and


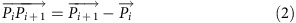


where 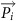 is the two-dimensional position at sample/stride *i*.

Change of heading (Δ*θ*_*i*_) was calculated from the angle between the two vectors:


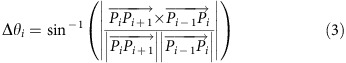


Angular velocity (*ω*_*i*_) was derived by dividing the change of heading by the time between mid-stride positions Δ*T*:


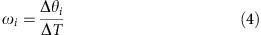


The tangential or fore-aft acceleration (*a*_t,*i*_) and centripetal acceleration (*a*_c,*i*_) were then computed from mid-stride speeds *v*_*i*_:


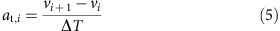



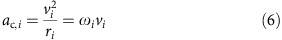


Negative values for tangential acceleration indicate deceleration. Positive and negative values for centripetal acceleration indicate right (+) and left (−) turns. Positive and negative centripetal acceleration values are presented separately to show if there was a preference for left-hand or right-hand turns.

### Improving accuracy through averaging

One important consideration when calculating heading, change of heading and heading angular velocity from position measurements is that accuracy will decrease as speed decreases. Although averaging over a stride and across strides markedly improves the accuracy, lower average speed values will still be less accurate. The noise present is of a level that does not unduly influence extreme values even at very low speeds^12^.

While validations carried out on the stride timing show that it is generally accurate, detection of an incorrect or spurious peak for end of stride would result in one stride duration being under or overestimated and the adjacent stride duration being affected in the opposite manner. This would introduce error in parameters that do not change smoothly through a stride, for example, acceleration, kinetic energy. We therefore applied a weighted average by taking the preceding and following stride into account:


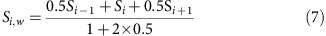


where *S* represents the parameter being weighted and *w* and *i* are the stride numbers.

This approach was used for tangential acceleration and centripetal acceleration, which were based on weighted stride speed and weighted heading rate.

### Run distance

Distances covered within individual runs were calculated by integration of the stride-averaged horizontal speeds over the duration of the run.

SDC duration was equal to the individual chase duration. In MDC, duration was defined as duration in which any dog participating in the MDC was still running (Fig. 4a). Duration and distance were calculated from Kalman-filtered GPS positions.

### Statistics

Three data sets were analysed containing runs (>3 ms^−1^; *n*=1,551), chases (<6 ms^−1^; *n*=1,119) and only successful chases (*n*=280; Supplementary Fig. 5). The comparison between the two different speed thresholds was carried out to compare robustness of results in relation with misclassification of possible non-hunting attempts.

Since tortuosity is infinite for a circle, we used the multiplicative inverse of tortuosity to test for significance.

A multivariate GLM was performed to assess the relationship between the number of dogs running (a single independent variable) and multiple dependent variables (maximum stride speed, run distance, run duration, maximum acceleration, maximum deceleration and a measure of tortuosity). Dog identity was also included as a dependent variable.

Using data for all chases, there was a significant multivariate effect for the combined dependent variables in respect of both the number of dogs (multivariate GLM, *n*=1,119, *λ*=0.814, F(30, 6454)=11.389, *P*=< 0.001) and individual (*λ*=0.79, F(30, 8085)=12.959, *P*=<0.001; Supplementary Table 2).

Correlation between the dependent variables was not equal (multivariate GLM, *n*=1,119, Box's *M*=2,114.078, F(609, 88485)=3.186, *P*=< 0.001) and with multiple variables being more than reasonably correlated (taken as 0.90 to −0.40) it was not appropriate to perform *post hoc* tests on these data. Owing to the high correlation between the dependent variables and the violation of the homogeneity of variance assumptions the outcome of the statistical analysis should be treated with caution. However, the significant increase/decrease of all parameters except tortuosity with the number of dogs in a DC is confirmed in the trend visualized in the violin plots (Fig. 3 and Supplementary Fig. 5).

To explore the influence of individual dogs on the outcome and to exclude the possibility that the significant change with group size is solely a result of the participation of a certain individual (and the increasing likelihood that this individual participates with increasing group size) we analysed the composition of the runs. We removed one individual, as well as all results from other individuals based on runs the removed individual participated in. The results of the exclusion of any of the six dogs are shown in Supplementary Fig. 6. The boxplots show that the general pattern observed using all trials is confirmed or even strengthened when removing individual dogs except for mean absolute heading rate and tortuosity.

*Maximum speed reliability*. The maximum stride speed of 19 ms^−1^ was reported for the following reasons: (1) all individuals achieved this speed at least once; (2) 19.4 ms^−1^ is the 99th percentile from maximum stride speeds from all runs; (3) using only maximum speeds from runs above 6 ms^−1^ the 99th percentile is 20.0 ms^−1^, taking the s.d. of 0.3 ms^−1^ for Kalman-filtered speeds (Supplementary Fig. 3b) and considering a maximum speed measurement error of three s.d.'s gives a maximum speed of 19 ms^−1^.

*Circular statistics*. To test whether the individuals have a preferred position within the pack when searching for food, we used the position of the individuals around the pack's centroid and tested for uniformity in distribution using the Hodges–Ajne test (Supplementary Fig. 7). Hodges–Ajne test is an alternative to the more commonly known Rayleigh test, without the restriction of unimodality or assumptions of the underlying distribution.

*Participation and initiation*. Binomial test of proportions on participation in MDCs and initiation of MDCs were conducted after the data were normalized for the number of days individuals participated in hunting (reduced due to the death of Kobe and the confinement to the den of Timbuktu) to test for significant difference between dominant and subdominant individuals.

### Cooperation

MDCs were assessed qualitatively to investigate the different trajectories recorded and in particular identify whether any chases involved higher levels of cooperation (coordination and collaboration). Cooperation can be defined simply as two or more individuals working together to achieve a common goal^14^. Under this definition, hunting as a pack and sharing the kill, regardless of the strategy used, is cooperative behaviour. African wild dogs, together with lions, are often described as a hunting team, taking on different but complementary roles during the chase itself. We were specifically interested in the presence or absence of these higher-level cooperative strategies during the chase. To provide greater insight into these complex behaviours, cooperation can be further divided into different levels based on the level of organization required (following Boesch and Boesch^14^ and modified by Bailey *et al*.^15^). The highest level, ‘collaboration', implies that individuals take on different roles during a hunt, for example, blocking and driving, and would be observed, for example, as individuals targeting the same prey at the same point, but from different directions. Some individuals may remain stationary or move to the sides, for example, to block the escape route of the prey. The next level down, ‘coordination', implies multiple dogs focus their attention on the same target point and relate to one another in space and time, for example, chasing as a group over changes in direction, or at least following similar relative trajectories, despite changes in running direction, likely in response to prey direction changes or chasing together until the final point where individuals may encircle the target. Differentiating between certain aspects of these behaviours can be difficult without knowing the movements of the prey. For example, driving prey from a different direction into other oncoming group members would imply collaboration, but splitting to encircle the prey would imply coordination. Both collaboration and coordination would imply that some higher level of cooperative strategy is used and all MDCs were visually assessed for evidence of either strategy.

### Spatial relationship between pack members

To discern a spatial pattern such as a spear or U formation in the search for prey, we used 10-s non-running GPS data collected for a period of 2 h each day at peak hunting times. Leading up to the start of a DC we calculated the centroid of the group at each point in time and determined the heading direction between two consecutive time points. Dogs further away than 150 m from the centroid were excluded, and the centroid position and the heading were recalculated. A rotation matrix was then applied to reorient individuals in the centroid heading direction. The lead dog was identified as the one furthest ahead in that direction. We then plotted three-dimensional heat maps with respect to the lead dog to discern movement pattern over time or related to speed. To discern changes in patterns during the 10 min up to the start of the chase, time points were averaged over 2-min periods and displayed. Changes in patterns with speed were displayed by binning time points in three speed categories: 0–1.5; 1.5–3; and >3 m s^−1^.

### Terrain analysis

We investigated whether vegetation varied between the start and end of a chase, using aerial photography images. A script was written in MATLAB that displayed a high-resolution aerial image (Google Earth) of a 50 × 50-m square centred on the beginning and end of each chase. An observer, who was not aware of the research question, manually scored the amount of tree, shrub (mopane), grassland and sand in each square by moving a slider with a mouse to record a value between 0 and 100. Data in each category were normalized to provide a percentage value for each square and then arcsine transformed^29^. The data were then categorized based on the highest percentage of cover in the field of view. The resulting four categories fit broadly with three out of five previously classified main habitat types found in the Delta^18^: grassland, mixed woodland and shrub.

To compare the proportion of different habitat types at the start and end of chases, Mann–Whitney *U*-tests were performed (as data did not meet the assumptions of normality; level of significance *P*<0.05, Supplementary Table 3).

## Additional information

**How to cite this article:** Hubel, T. Y. *et al*. Additive opportunistic capture explains group hunting benefits in African wild dogs. *Nat. Commun.* 7:11033 doi: 10.1038/ncomms11033 (2016).

## Supplementary Material

Supplementary Information Supplementary Figures 1-7, Supplementary Tables 1-3, Supplementary Notes 1-2 and Supplementary References.

Supplementary Movie 1 Path and speed of all 24 MDCs of 5 dogs running overplayed on satellite image. Origin at location where first dog started running; start of run indicated by circle; end of run indicated by cross; location of dog five minutes after end of run indicated by star (if within field of view) and written in brackets.

Supplementary Movie 2 Examples for typical behavior such as an unsuccessful DC (39, 93, 285, 209), successful DC (141, 182, 191, 281) and rally/greet (142, 1247, 1715). Animation shows path of multiple dogs running based on GPS data. Origin at location where first dog started running; path extended to encompass 10 minute GPS positions before (circle) and after the run (cross). Distance between end of run and 5 minutes after position written in brackets (legend) and marked with a star. Runs indicated by high sample rate (more solid lines). Individuals not running, but present, indicated by grey marker trace. Note that GPS sample rate can differ depending on time of the day, therefore the density of points before and after the run can vary between trials. Kills often indicated by cross and star in same location after run.

## Figures and Tables

**Figure 1:**
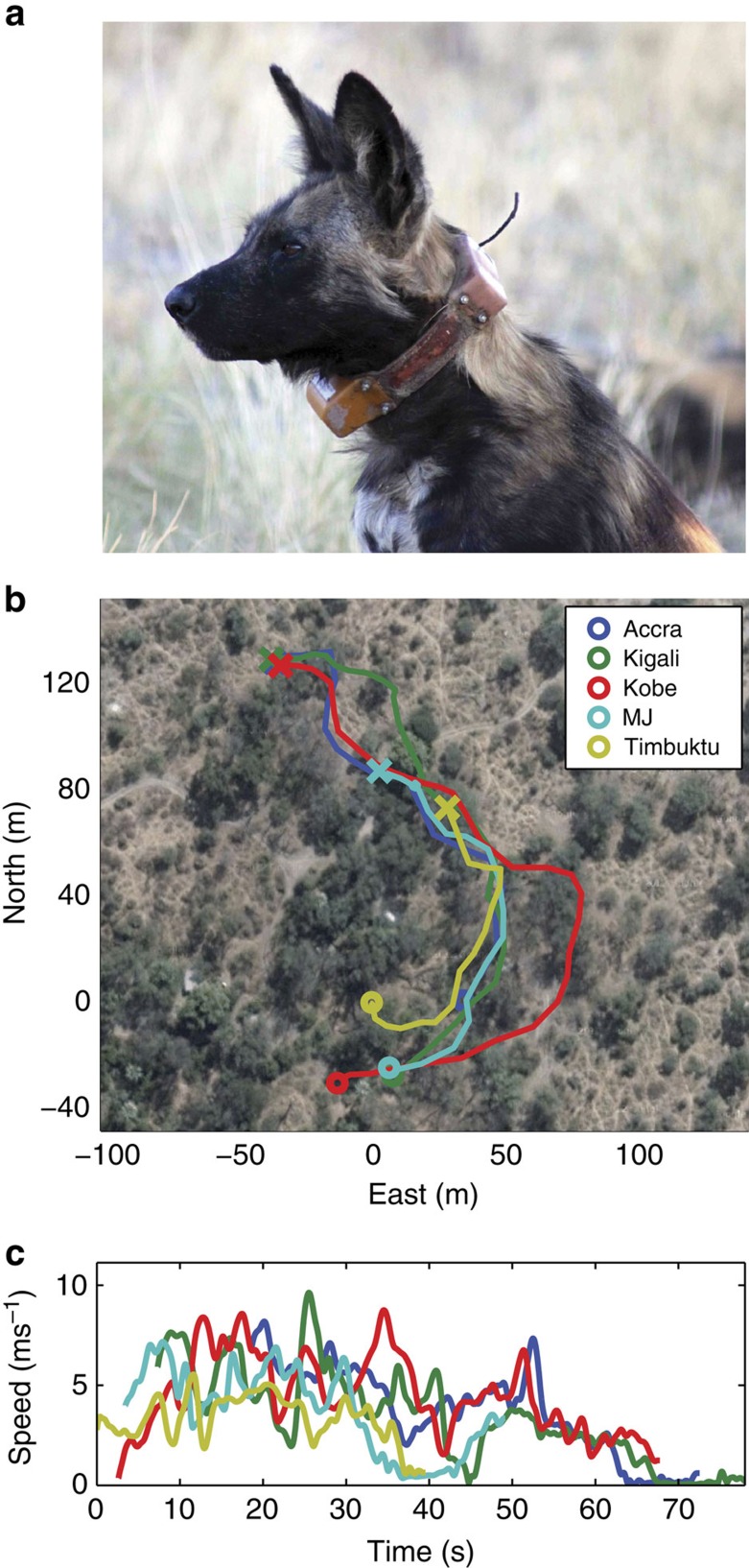
GPS collar and traces. (**a**) African wild dog with collar. (**b**) Example GPS trace for unsuccessful MDC involving five individuals, origin at MDC start position, run start (circle) and run end (cross). (**c**) Speed profile for each dog in **b**.
