# Supplementary material for: Additive opportunistic capture explains group hunting benefits in African wild dogs
Source: Nat Commun. 2016 Mar 29;7:11033. doi: 10.1038/ncomms11033 (PMC4820541; doi:10.1038/ncomms11033)
Supplement: Supplementary Information — Supplementary Figures 1-7, Supplementary Tables 1-3, Supplementary Notes 1-2 and Supplementary References. [file ncomms11033-s1.pdf]

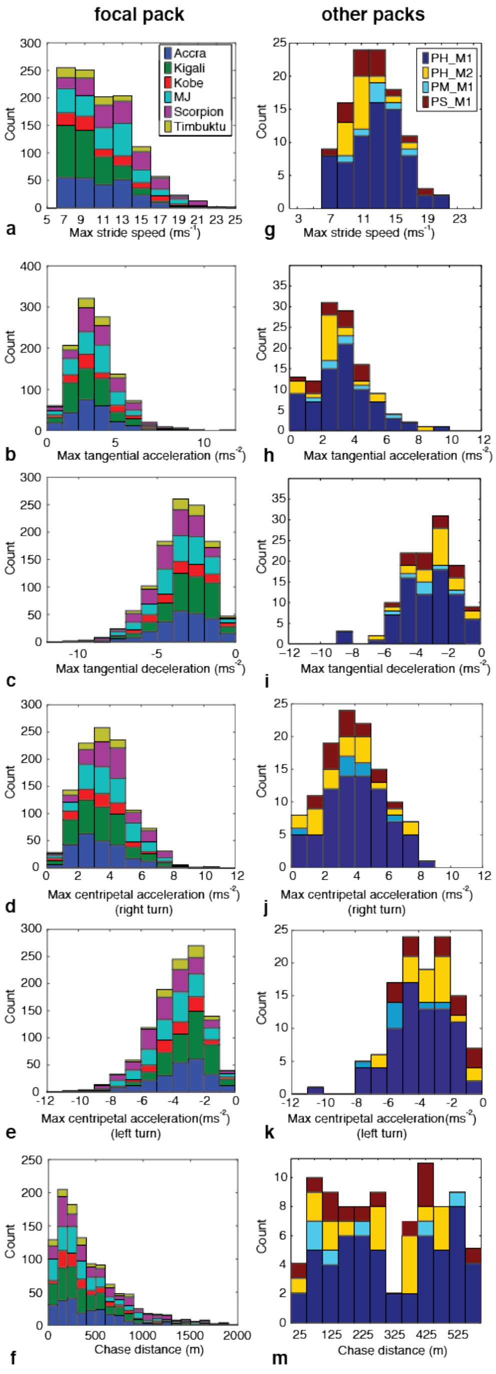

Supplementary Fig. 1: Comparison of chase parameters for focal pack (a-f, n=1119) and for 4 dogs from 3 other packs (g-m, n=107). (a,g) Maximum stride speed, (b,h) maximum tangential acceleration, (c,i) maximum tangential deceleration, max centripetal acceleration (d,j) right and (e,k) left turn, and (f,m) distance covered while chasing, colour coded by individual dog to highlight individual contribution to histogram. Note: collar triggered into chase mode based on IMU (focal pack) or GPS (other packs) threshold. GPS triggering was less sensitive, especially at low speeds.

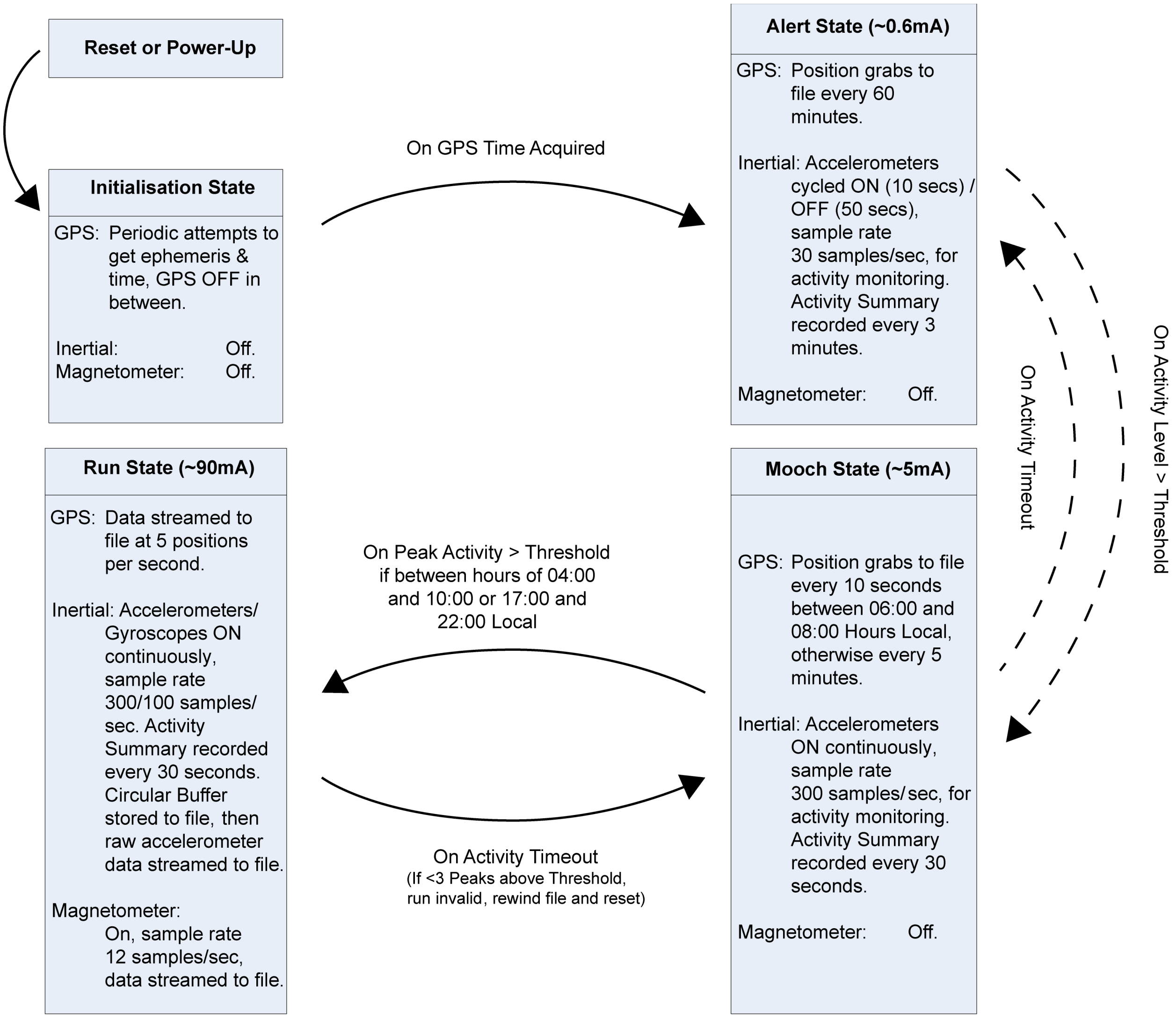

Supplementary Fig. 2: Wildlife collar state and transition diagram as used during the majority of data collection time 21 May – 31 Aug. 2012. Collar settings were optimised during the first few weeks after deployment on 13 April 2012, most notable was the eradication of pre-buffering in favour of an extending time period when collars were allowed to go into chase state on 26. April 2012 and the switch from mooch state with position grabs every 10 seconds from 18:00-20:00 to 6:00-8:00 on 21 May 2012.

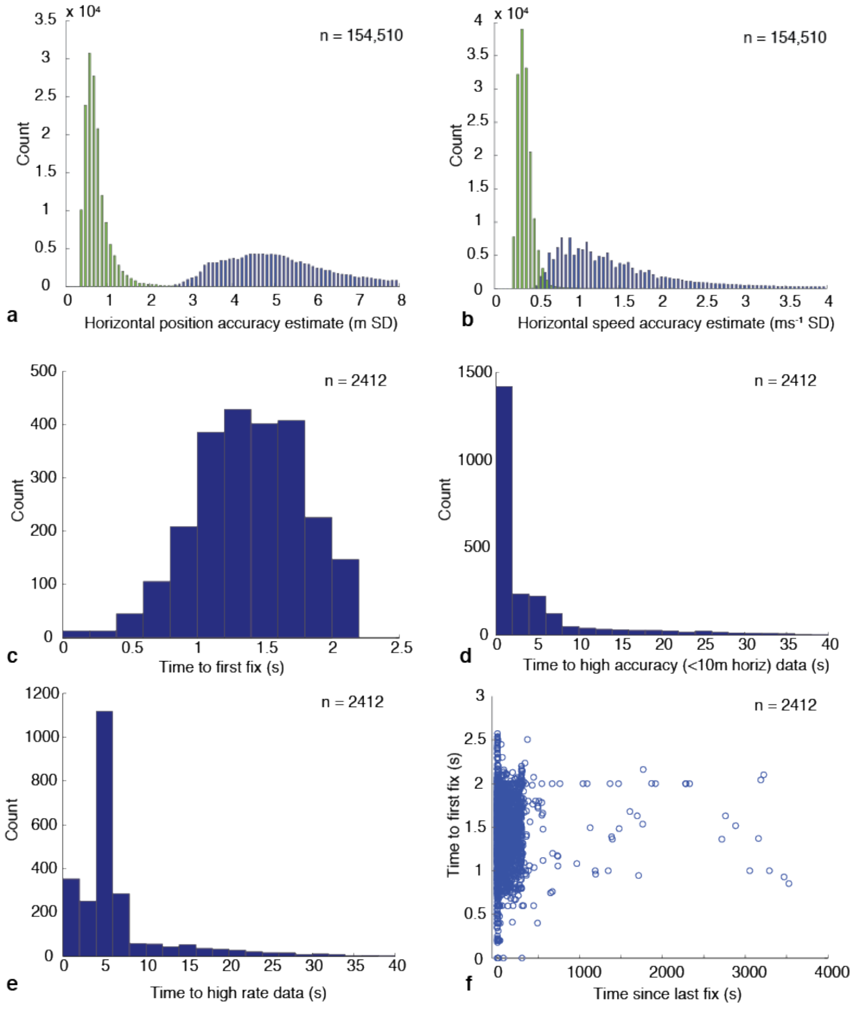

Supplementary Fig. 3: Summary of GPS performance in the field during the study. (a,b). Horizontal position accuracy standard deviation estimate blue, from the GPS module for the stand alone GPS data, green from the Kalman smoother once GPS and IMU data have been fused. The estimation algorithm inherently gives error covariances of each parameter for each sample based on measurement error calculated for each fix by the GPS module and IMU errors from lab testing. (a) GPS horizontal position error (median stridewise SD) was reduced by data fusion from 5.84±2.71 m (pure GPS data) to 0.72±0.36 m in the smoothed solution. (b) Equivalent plot to (a) for horizontal speed. Speed error was reduced from 1.49±0.79 ms^{-1} to 0.36±0.09 ms^{-1}, i.e. data fusion delivers a four to eight fold improvement in accuracy. (c) Time from triggering the GPS module at the start of a hunt to receiving a valid position/speed fix; this was almost always (92.8% triggers) within two seconds (d) Time from triggering the GPS module at the start of a hunt to receiving a high accuracy (horizontal error<10 metres) fix. 69.1% triggers were within two seconds of trigger (e) Time from trigger to receiving high rate (5 Hz) position data 17.2% triggers within two seconds, 52.1% within five seconds and 95.4% within seven seconds. (f) Time from trigger to high rate data against time since last fix (testing the refresh rate required for rapid startup). N=2412 for this figure because startup data are logged for every trigger not just valid runs.

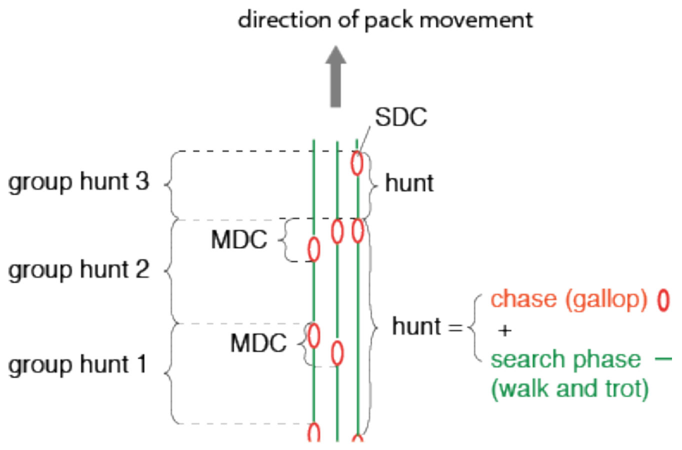

Supplementary Fig. 4: Visualisation of terms used in the multi-level analysis of individual chases and hunts as well as showing pack dynamics.

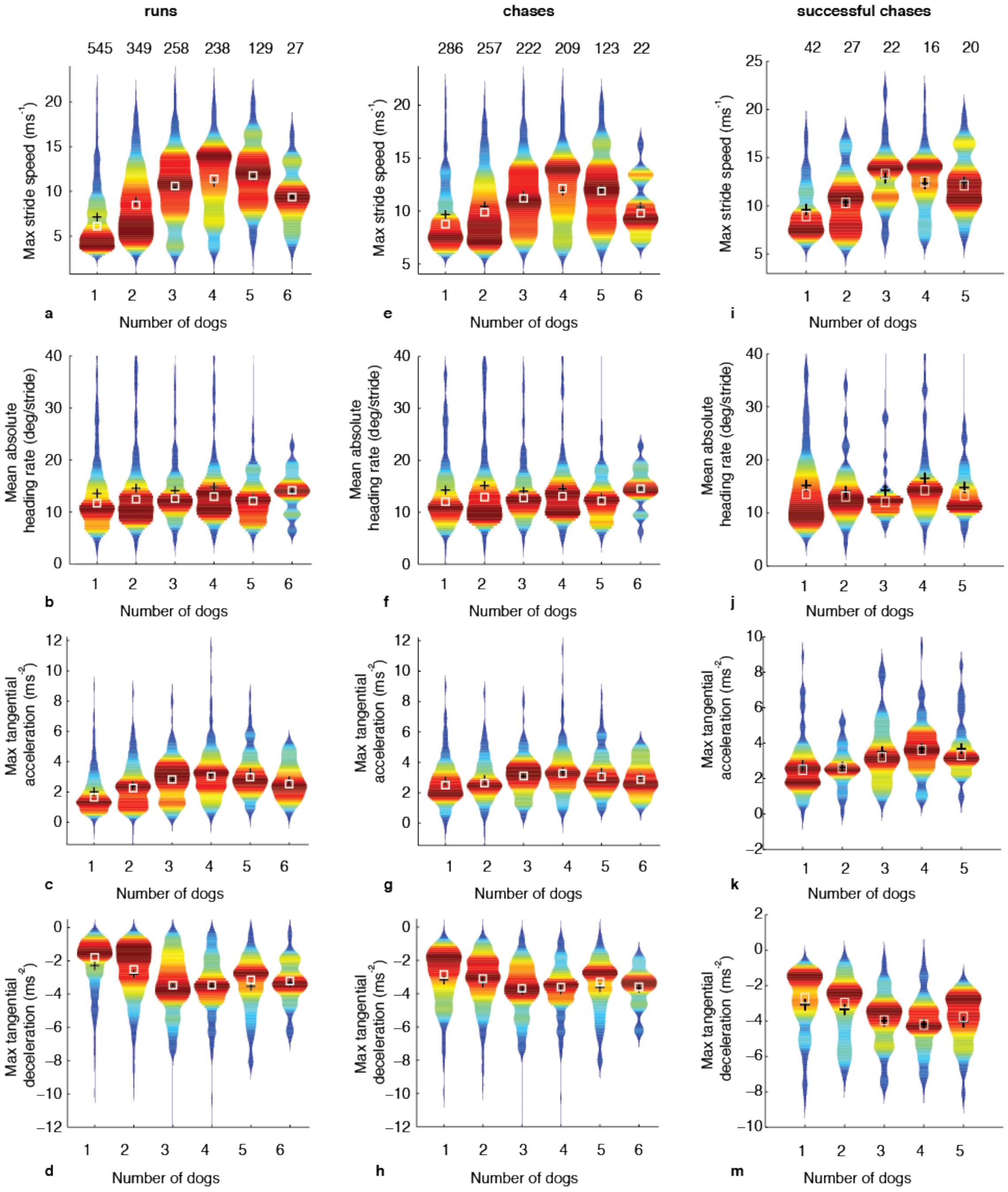

Supplementary Fig. 5: Comparison of subset of run parameters versus number of dogs running simultaneously for different datasets. Results are displayed as violin plots (combining box plot and kernel density plot) showing the density distribution of the values; with each histogram normalised to the same maximum bin width in order to compare distribution shape. Mean (black cross); median (white box). The figure shows the consistency in the results by comparing datasets with different speed thresholds, (a-d) runs; n=1551 (e-h) chases, n=1119, and (i-m) successful chases, n=127 (c). The total number of values contributing to each violin plot, is at the top of each the violin plots and applies to all plots below.

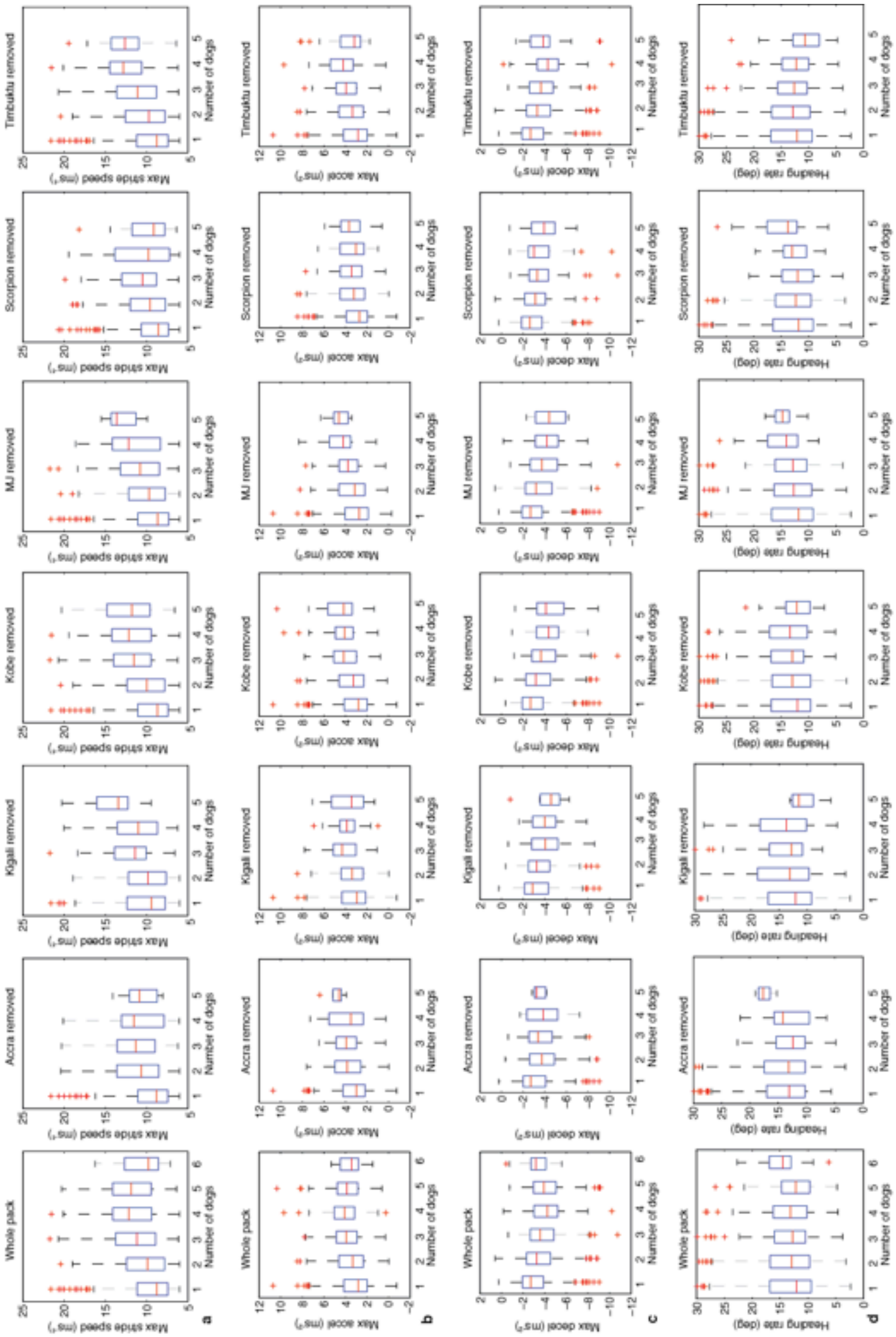

Supplementary Fig. 6: Boxplots for selected run parameters for differ datasets. (a) Maximum stride speed, (b) maximum tangential acceleration, (c) maximum tangential deceleration, (d), mean absolute heading rate versus group size displayed for all data and 6 subsets with one of the individuals and all MDCs the individual participated removed one at a time. All data presented are from chases.

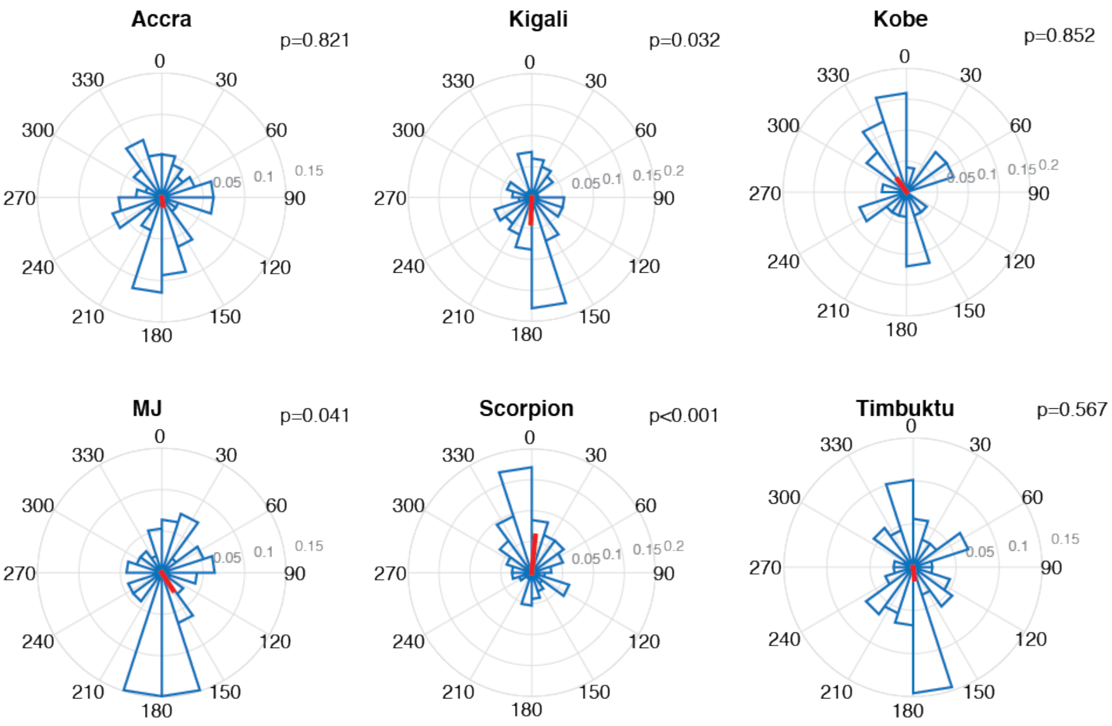

Supplementary Fig. 7: Circular distribution of the individuals around the packs centroid. Position was averaged over the 10 minutes travel prior to the start of an MDC. *p*-values displayed are based on the Hodges-Ajne test at a significance level of 0.05.

Supplementary Table 1: Information regarding individual dogs in the pack and darting information.

Individual:	Accra	Kigali	Kobe	MJ	Scorpion	Timbuktu
**Sex:**	Female	Female	Male	Male	Male	Female
**Age:**	3 years (born 2009)	4 years (born 2008)	3 years (born 2009)	4 years (born 2008)	?	3 years (born 2009)
**Rank:**	sub-ordinate	sub-ordinate	dominant	sub-ordinate	?	dominant female
**Body condition (very poor 1 to excellent 5):**	5 (excellent)	4	5 (excellent)	5 (excellent)	5 (excellent)	5 (excellent)
**Injury?**	No	Healed broken leg (front left) but remaining slight limp and a kiwi sized ball of fluid/blood on the back of the upper leg near the break.	couple of small infection spots (ticks) and slight wound on the top right forelimb	No	No	No
**Mass (kg): (empty stomach estimate)**	25-26	26	~ 29-30	~ 32	~ 30	~ 29
**Hindlimb length (mm): (standing posture)**	542	561	528	564	590	528
**Forelimb length (mm): (standing posture)**	480	476	485	570	490	440
**Back length (mm): (top of neck to base of tail)**	822	820	880	950	910	810
**Drugs:**	55 mg xylazine; 50 mg ketamine; 1.2 mg atropine	55 mg xylazine; 50 mg ketamine; 1.2 mg atropine	55 mg xylazine; 50 mg ketamine; 1.1 mg atropine	55 mg xylazine; 50 mg ketamine; 1.1 mg atropine	55 mg zylazine; 50 mg ketamine; 1.1 mg atropine	55 mg xylazine; 50 mg ketamine; 1.2 mg atropine
**Time antidote delivered after darting:**	46 min; yohimbine 4 mg	51 min; yohimbine 4 mg	45 min; 5.5 mg antisedan	49 min; antisedan 5.5 mg	44 min; yohimbine 4 mg	44 min; yohimbine 4 mg

Supplementary Table 2: Results of multivariate GLM performed to assess the relationship between the number of dogs running (a single independent variable) and multiple dependent variables (maximum stride speed, run distance, run duration, maximum acceleration, maximum deceleration, maximum centripetal acceleration (left and right turns) and a measure of tortuosity). Individual dog identity was also included as a dependent variable.

Effect		Value	F	Hypothesis df	Error df	p-value
Intercept	Pillai's Trace	0.939	4141.176[b]	6	1613	<0.001
	Wilks' Lambda	0.061	4141.176[b]	6	1613	<0.001
	Hotelling's Trace	15.404	4141.176[b]	6	1613	<0.001
	Roy's Largest Root	15.404	4141.176[b]	6	1613	<0.001
individual	Pillai's Trace	0.215	12.086	30	8085	<0.001
	Wilks' Lambda	0.791	12.959	30	6454	<0.001
	Hotelling's Trace	0.256	13.755	30	8057	<0.001
	Roy's Largest Root	0.224	60.383[c]	6	1617	<0.001
number of dogs	Pillai's Trace	0.191	10.703	30	8085	<0.001
	Wilks' Lambda	0.814	11.389	30	6454	<0.001
	Hotelling's Trace	0.224	12.011	30	8057	<0.001
	Roy's Largest Root	0.196	52.786[c]	6	1617	<0.001

a. Design: Intercept + individual + total number of dogs running

b. Exact statistic

c. The statistic is an upper bound on F that yields a lower bound on the significance level.

Supplementary Table 3: (a) Results from Mann-Whitney U test for difference in habitat types between the beginning and end of run. h is 1 if the test rejects the null hypothesis (no difference) at the 5% significance level. (b) Results from Mann-Whitney U test for difference in percentage of the different habitat types at the start and end of a run for successful vs. non-successful chases.

a

Category	Mean percentage of cover		Standard deviation		Results from Mann-Whitney U test for differences in habitat type between the start and end of a run			
	Start	End	Start	End	Mann-Whitney U	Z	p	h
Mixed woodland	23.98	23.3	19.19	18.65	1611459.5	-0.903	0.367	0
Mopane (scrub)	10.9	11.77	12.45	13.03	1573413	-2.123	0.034	1
Grassland	59.42	59.31	21.7	21.09	1633934	-0.188	0.851	0
Sand	5	4.9	10.12	9.98	1638354.5	-0.052	0.959	0

b

	Results from Mann-Whitney U test for differences between successful and unccussesful runs with habitat type			
**Start of run**	**Mann-Whitney U**	**Wilcoxon W**	**Z**	**p**
Mixed woodland	113010.5	123021.5	-0.793	0.428
Mopane (scrub)	114049	124060	-0.622	0.534
Grassland	115605.5	1510890.5	-0.357	0.721
Sand	113636	123647	-0.742	0.458
**End of run**				
Mixed woodland	115554	125565	-0.366	0.714
Mopane (scrub)	108639.5	118650.5	-1.533	0.125
Grassland	115628	125639	-0.353	0.724
Sand	113848	123859	-0.703	0.482

Supplementary Note 1

Wild dog hunting (record of a kill observed on 21st May 2012 by JPM).

Trial 182 (17:40 on 21st May 2012), which involved 5 dogs running, was also directly observed in the field (by JPM). During this event the pack was followed prior to entering a tree line where a herd of impala were present. The individual MJ was observed breaking out of the tree line and chasing a single juvenile male impala through open grassland and into mixed scrub where a kill was made. The chase and kill were performed by MJ alone; no other dogs were visible at the time. Post-kill two other dogs joined to feed (Timbuktu and Scorpion); no other member of the pack fed from this kill. However, once the three dogs had finished feeding they moved to another location in the tree line (to the North-East of original kill) where a second kill (also a juvenile male impala) had also been made and the remaining three dogs were feeding. Once the pack had finished feeding at the second kill they rested very briefly before moving off.

From the animation it can be seen that four of the dogs (MJ, Kobe, Accra and Kigali) began running at approximately the same time (within 30 seconds of each other), from a similar location. Accra and Kigali ran in one direction and MJ and Kobe in another (likely targeted on different prey individuals). Accra made a kill first and at this point Kobe (the dominant male) stopped chasing in the same direction as MJ and re-routed to the first kill to feed. MJ continued to chase his prey individual alone and also made a kill. Scorpion probably chased another prey individual, but was not successful and re-routed to feed at MJ's kill.

This scenario reflects what appears to happen in the majority of MDCs recorded in this study, where the pack travels together until flushing a herd of prey, upon which individual dogs independently pursue prey. One or more dogs may kill in such an event. Such a strategy is a cooperative behaviour in that by hunting as a group to make simultaneous kills they increase the chances of at least one kill being made, compared to if just one dog went out to hunt on its own. However, such a strategy does not appear to involve higher-level collaboration, where multiple predators focus on a single prey item and take on different roles in order to capture that one prey individual successfully[1], refined by[2]. This is often the scenario pictured when cooperative hunting is described in large predators such as lions. It does not, however, mean that African wild dogs are not capable of such behaviours, or that they do not benefit from them in certain circumstances, rather that such a strategy is less beneficial for the current pack studied than chasing individual prey. The strategy exhibited is likely to be related to a combination of factors including common prey type (small-medium sized prey such as impala can be captured by single dogs), habitat (dense habitat may restrict the ability to coordinate group movements) and pack size (a small pack does not need to hunt the larger prey e.g. wildebeest, which may require more collaborative strategies. These prey are also not present in very large herds in the study area which tends to encourage collaborative hunting e.g. dogs distract a mother wildebeest while others hunt the calf[3].

Even though this MDC was recorded by human observers, it was impossible for the observers to locate and record the movements of all the individuals in the pack during the chase (only one individual was directly observed chasing). In fact, during three months of observations in the field only one kill was observed directly, and only a handful of kills were noted over the entire 6 month period (~ 11 kills, mostly recorded when individuals were

located on a kill). This highlights how difficult it is to directly observe and record group hunting strategies in this species, particularly in the dense habitat found throughout much of their range. Although information such as prey type is rarely available for these data, we have data on a much larger number of chases over a shorter time period than previously available, in a habitat where it is not possible to record such data by human observation. Much of the previous information about hunting in these species has come from direct human observation in more open habitats. While it is useful to build up observational data sets, they may often miss the types of hunts that occur in different habitats, or occur when the light levels are poor and observers are not following them. The completeness of the predator hunting data captured for the first time here provide us with a broader and more detailed image of wild dog hunting strategies.

Supplementary Note 2

Participation and initiation in MDCs (Fig. 5 extended explanation)

Figure 5 shows how often individual dogs run in SDCs and MDCs normalised by number of days the individuals were active, taking into account that Kobe died and Timbuktu was denning for part of the 118 days of data collection.

Figure 5a accounts for all runs. Noticeable is the high number of single runs conducted by Kigali. Field observation showed that Kigali walked with a slight limp due to a healed broken front leg and sometimes fell behind when walking and trotting with the pack. The old injury did not seem to hinder Kigali while running and the high number of runs can be attributed to her catching up with the pack. Fig. 5b shows only chases, and the reduced difference in SDCs between Kigali and the other pack members confirms that most of her SDCs are low speed runs. Both figures confirm the notion that the dominant pair (Kobe and Timbuktu) conducted fewer runs than the rest of the pack, which could be attributed to the fact that the alpha pair have first feeding rights after the pups have finished. Subdominant adults are the last to feed on the remains and if the kill was small have to leave in search for another kill[4]. Fig. 5d-f show the percentage for which each individual initiated the MDC (2 dogs and more) based on the time each collar triggered into 'run state'. Most times the subdominants Accra, Kigali and MJ started running first. The contributions change little between values for all runs (Fig. 5d), chases (Fig. 5e) successful chases (Fig. 5f). Again data were normalised based on the number of days each individual was able to go hunting.

Supplementary References

1 Bailey, I., Myatt, J. P. & Wilson, A. M. Group hunting within the Carnivora: physiological, cognitive and environmental influences on strategy and cooperation. *Behav Ecol Sociobiol* **67**, 1-17, doi:10.1007/s00265-012-1423-3 (2013).

2 Boesch, C. & Boesch, H. Hunting behavior of wild chimpanzees in the Taï National Park. *American Journal of Physical Anthropology* **78**, 547-573, doi:10.1002/ajpa.1330780410 (1989).

3 Stander, P. E. Cooperative hunting in lions: the role of the individual. *Behav Ecol Sociobiol* **29**, 445-454 (1992).

4 Burrows, R. *A Guide to the African Wild Dog*, <http://www.africanwilddogwatch.org/library/documents/AWDW0001.pdf>
